# Expression Profiling Coupled with *In-silico* Mapping Identifies Candidate Genes for Reducing Aflatoxin Accumulation in Maize

**DOI:** 10.3389/fpls.2017.00503

**Published:** 2017-04-06

**Authors:** Ramesh Dhakal, Chenglin Chai, Ratna Karan, Gary L. Windham, William P. Williams, Prasanta K. Subudhi

**Affiliations:** ^1^School of Plant, Environmental, and Soil Sciences, Louisiana State University Agricultural CenterBaton Rouge, LA, USA; ^2^Department of Agronomy, University of FloridaGainesville, FL, USA; ^3^USDA-ARS Corn Host Plant Resistance Research UnitMississippi State, MS, USA

**Keywords:** aflatoxin, gene expression, marker-assisted breeding, QTL, SSH library, reverse northern, *Zea mays*

## Abstract

Aflatoxin, produced by *Aspergillus flavus*, is hazardous to health of humans and livestock. The lack of information about large effect QTL for resistance to aflatoxin accumulation is a major obstacle to employ marker-assisted selection for maize improvement. The understanding of resistance mechanisms of the host plant and the associated genes is necessary for improving resistance to *A. flavus* infection. A suppression subtraction hybridization (SSH) cDNA library was made using the developing kernels of Mp715 (resistant inbred) and B73 (susceptible inbred) and 480 randomly selected cDNA clones were sequenced to identify differentially expressed genes (DEGs) in response to *A. flavus* infection and map these clones onto the corn genome by *in-silico* mapping. A total of 267 unigenes were identified and majority of genes were related to metabolism, stress response, and disease resistance. Based on the reverse northern hybridization experiment, 26 DEGs were selected for semi-quantitative RT-PCR analysis in seven inbreds with variable resistance to aflatoxin accumulation at two time points after *A. flavus* inoculation. Most of these genes were highly expressed in resistant inbreds. Quantitative RT-PCR analysis validated upregulation of PR-4, DEAD-box RNA helicase, and leucine rich repeat family protein in resistant inbreds. Fifty-six unigenes, which were placed on linkage map through *in-silico* mapping, overlapped the QTL regions for resistance to aflatoxin accumulation identified in a mapping population derived from the cross between B73 and Mp715. Since majority of these mapped genes were related to disease resistance, stress response, and metabolism, these should be ideal candidates to investigate host pathogen interaction and to reduce aflatoxin accumulation in maize.

## Introduction

*Aspergillus flavus* is an endemic fungus that is responsible for ear rot disease and aflatoxin contamination in maize (Widstrom, 1996). Aflatoxins are secondary metabolites produced after *A. flavus* infection and have carcinogenic, immunosuppressive, and hepatotoxic properties. Consumption of aflatoxin contaminated grains can be harmful for both human and domestic animals. Aflatoxins are the most widely studied mycotoxins due to its ability to suppress human and animal immune systems and induce liver cancer (Hawkins et al., 2015). Aflatoxin contamination drastically reduces grain value leading to significant economic loss worldwide. Many countries around the world have established rules and regulations for minimizing aflatoxin contamination in foods and feed (FAO, 2004). The U.S. Food and Drug Administration (USFDA, 2008) has banned the interstate commerce and consumption of the maize grain contaminated with more than 20 ng/g-1 for aflatoxin.

*A. flavus* can readily attack, and colonize various economically important crops such as cotton, pecans, and peanut in addition to corn (Diener et al., 1987). Researchers initially focused on preventing post-harvest accumulation of aflatoxin through management of storage condition (Trenk and Hartman, 1970). Subsequently, research efforts were directed for identification and utilization of host plant resistance for the development of new inbreds (Diener et al., 1987) because it is an effective and sustainable approach to minimize *A. flavus* infection and aflatoxin accumulation in maize. In recent decades, more emphasis has been given for these activities and resistant germplasm have been identified (William, 2006; Williams and Windham, 2012; Warburton et al., 2013). Transferring resistance to commercial hybrids has been a challenge due to the quantitative nature of resistance, low heritability, inconsistent occurrence of disease, and G × E interaction (Dolezal et al., 2014). Most resistant lines are agronomically inferior and are not suitable for developing hybrids (Brown et al., 1999). No inbred or hybrid has been reported to have complete resistance to *A. flavus* infection (Abbas et al., 2006). Previous studies have shown moderate to low contribution of genetic component on resistance to aflatoxin accumulation (Warburton et al., 2009, 2011). Therefore, introgression of multiple resistant quantitative trait loci (QTL) to susceptible, adaptive, and high yielding lines through marker assisted breeding (MAB) may be an effective method to breed aflatoxin resistant maize varieties. However, the lack of reliable molecular markers and limited understanding of host-plant resistance mechanisms hindered the progress in introgression of resistant QTL/genes to the commercially acceptable and adapted inbreds.

QTL mapping studies have been conducted using diverse mapping populations (Paul et al., 2003; Brooks et al., 2005; Bello, 2007; Warburton et al., 2015). QTL with variable phenotypic effects were identified on chromosomes 4, 5, 8, and 10 (Brooks et al., 2005; Warburton et al., 2009, 2011; Willcox et al., 2013; Yin et al., 2014). Meta-analysis using various populations further confirmed QTL on chromosome 4 and QTL in bin 4.08 was confirmed using near-isogenic lines (Mideros et al., 2014). Despite continuous efforts, the identification of QTL with consistent large phenotypic effects has been rarely identified.

Although, several mapping studies have been performed for elucidating the genetics of aflatoxin resistance, investigations on molecular mechanisms of host-plant resistance and host-pathogen interactions are limited. Resistance to reduce *A. flavus* infection in maize requires involvement of many genes (Brooks et al., 2005; Kelly et al., 2012). Several studies have identified resistance genes with various functions in maize kernels after inoculation (Doehlemann et al., 2008; Alessandra et al., 2010). Upregulation of many stress, signal transduction, and pathogenesis related genes in resistant germplasm (Jiang et al., 2011; Luo et al., 2011; Kelly et al., 2012) indicated that these genes might be involved in resistance response to *A. flavus* infection in maize.

Suppression subtractive hybridization (SSH) is a cost effective and widely used method for generating subtracted cDNA libraries (Diatchenko et al., 1996). It can identify low-abundance differentially expressed genes (DEGs) between different genotypes, treatments, biotic/abiotic stress, and environmental conditions (Rebrikov et al., 2004). In maize, DEGs in response to *R. solani* and drought tolerance have been identified using this method (Li et al., 2007; Zhang et al., 2012). There are no studies that integrated multiple approaches such as identification of DEGs, their validation in various germplasm, and co-localization in QTL regions for resistance to aflatoxin. The objectives of this study are to identify DEGs using SSH, analyze their expression, validate in a set of resistant and susceptible inbreds, localize the DEGs on the linkage map constructed in an *F*_2:3_population of the cross B73 × Mp715 (Dhakal et al., 2016), and identify potential candidate genes involved in host-plant resistance for future studies.

## Materials and methods

### Field evaluation of germplasm

Seven maize inbred lines with different levels of resistance and susceptibility to aflatoxin accumulation were selected for the field experiment. The resistant inbreds were Mp715, Mp719, Mp420, Mp313E, and Mo18W whereas B73 and Va35 were susceptible to *A. flavus* infection (William, 2006; Gardner et al., 2007). The inbreds were planted in 2013 and 2014 in a randomized complete block design with three replications at the Louisiana State University AgCenter Central Research Farm, Baton Rouge (30°20′51″ N, 91°10′14″ W). Primary ears were inoculated 2 weeks after mid-silk (50% of the plants in a plot had silk exposed) using the side-needle technique with 3.4 mL of inoculum containing 3 × 10^8^ *A. flavus* conidia/mL. A 50 g kernel sample from each plot was used for aflatoxin quantification after harvesting. The VICAM AflaTest (VICAM, Watertown, MA) was used to quantify the aflatoxin concentration in each sample (Warburton et al., 2011). Aflatoxin concentration data were averaged for each inbred for each replication and natural log-transformed before the analysis to reduce non-normality. Analysis of variance (PROC MIXED) was carried out using SAS 9.3 (SAS Institute, 2010).

### Preparation of fungus culture

The *A. flavus* strain (NRRL 3357) was obtained from the ARS Culture Collection, Bacterial Foodborne Pathogens and Mycology Research Unit, USDA-ARS, Peoria, IL. It was grown overnight in a potato dextrose broth, and then grown on sterile maize-cob grits (size 2040, Grit-O-Cob, The Andersons Co., Maumee, OH) in a 500 ml flask for 3 weeks at 28°C. Conidia from each flask was washed from the grit with 500 ml of sterile water, which contained 20 drops of Tween 20/liter and was filtered through four layers of cheese cloth and stored at 4°C.

### Suppression subtraction hybridization (SSH) library preparation

Primary ears of inbreds, B73 (susceptible) and Mp715 (resistant), were inoculated with 3.4 mL fungal solution (3 × 10^8^ conidia/mL) using a side-needle technique after mid-silk. Inoculated and non-inoculated ears from each inbred were collected 2 and 3 weeks after inoculation. The harvested ears were immediately frozen in liquid nitrogen. The kernels around the point of inoculation were carefully separated and stored in −80°C for use in the experiment.

Total RNA from the inoculated and non-inoculated ears of each inbred was extracted. A modified SDS/Trizol RNA extraction was followed in order to isolate high quality RNA (Wang et al., 2012). RNA quality and integrity was checked in a 1.0% formamide-denaturing agarose gel. RNA quantification was measured by a ND-1000 spectrophotometer (Thermo Fisher Scientific, Wilmington, USA). Total RNA was treated with *DNAse*I and stored at −80°C. Messenger RNA was isolated using a PolyTract mRNA isolation kit (Promega, Madison, USA). Two micrograms of poly (A^+^) mRNA was used for the synthesis of double stranded DNA. Suppression subtraction hybridization (SSH) was carried out using a cDNA subtraction kit (Clontech, Wisconsin, USA) following the instructions of the manufacturer. A forward SSH library was prepared by using cDNA synthesized from the developing kernel of the inoculated resistant line Mp715 as the tester and cDNA of the inoculated susceptible line B73 as the driver. pGEM-T Easy vector (Promega, Madison, USA) was used to clone the subtracted cDNA fragments and then the cDNA clones were transformed into DH5α cells of *E. coli*. These transformed cells were grown on LB medium plates containing 100 mg/L ampicillin, 1 mM IPTG, and 80 mg/L X-gal at 37°C overnight. Positive colonies were selected and grown in LB medium containing ampicillin (50 mg/L) in a 96-well plate for 5 h at 37°C and stored at −80°C with 30% glycerol.

### Sequencing and classification of differentially expressed clones

Four hundred and eighty clones were randomly selected and sequenced. Sequencing of selected cDNA clones was conducted using the vector specific T7 and SP6 primers at the University of Washington High Throughput Genomics Unit. The BLASTX program in the National Center for Biotechnology Information (NCBI) (http://blast.ncbi.nlm.nih.gov/Blast.cgi) was used to identify sequences in a non-redundant database that are homologous to the sequences obtained from the SSH (Altschul et al., 1997). The *E*-value was examined to determine the significance of these matches. These genes were classified into different functional categories based on their functions using information obtained from NCBI, Gene Ontology (http://geneontology.org/), UniProt (http://www.uniprot.org), and related literature.

### Differential screening of the SSH library

The selected clones were grown overnight at 37°C with gentle stirring in LB medium for the isolation of cDNA inserts. Plasmid DNA was isolated using plasmid miniprep kit from Qiagen (Valencia, CA, USA). The cDNA inserts were amplified using vector specific T7 and SP6 primers flanking both sides of the inserts. Each PCR reaction was carried out in a reaction volume of 25 μl with the following composition: 11.8 μl of nuclease free water, 2.5 μl of 10X buffer, 2.5 μl of magnesium chloride (25 mM), 2.5 μl of dNTPs (2.0 mM), 1.25 μl each of T7 and SP6 primers (50 ng/μl), 0.2 μl of Promega Taq (5 U/μl), and 3.0 μl of template DNA (50 ng/μl). PCR was performed according to the following thermal cycling parameters: 95°C for 4 min; 95°C for 45 s, 55°C for 45 s, 72°C for 1 min, 35 cycles; 72°C for 5 min final extension. The PCR products were electrophoresed on a 1.2% agarose gel to confirm the size of inserts.

The PCR products containing cDNA inserts were concentrated using Eppendorf Vacufuge (Hamburg, Germany) at 4°C for 30 min. The concentrated products were denatured with 0.6 M NaOH before blotting. Five microliters of concentrated PCR product of each clone were blotted in duplicate to Hybond-N^+^ (Amersham Biosciences Corp., Piscataway, NJ, USA). Each blot consisted of 48 PCR products, which included 46 cDNA clones, actin (positive control), and water (negative control). After blotting, the membranes were allowed to air-dry at room temperature. The air-dried membranes were neutralized with Tris-HCl (1.5 M NaCl, 0.5 M Tris-HCl, pH 8.0) for 5 min. They were rinsed with SSC Tris-HCl solution (2x SSC, 0.2 M Tris-HCl, pH 8.0) for 30 s with gentle shaking and then dried. The membranes were carefully wrapped using plastic wrap and cross-linked with UV-ray for 1 min. cDNA probes were prepared from both inoculated and uninoculated RNA using an iScript cDNA synthesis kit (Bio-Rad, CA, USA). The probe labeling, prehybridization, hybridization, membrane washing, and detection were performed using ECL Direct Nucleic Acid Labeling, and Detection Systems (Cat # RPN 3000, GE Healthcare, Pittsburg, USA). After overnight exposure, X-ray films were developed. DEGs were selected on the basis of signal strength of the clones with different probes.

### Semi-quantitative reverse transcriptase PCR (RT-PCR) in maize germplasm

The tissues were collected from each germplasm from the control and *A. flavus* inoculated plants. To study changes in gene expression, two developing ears from each germplasm were harvested 24 h, and 48 h after inoculation along with the control and immediately placed in liquid nitrogen. The developing kernels were then separated and stored at −80°C. Total RNA was isolated from seven inbreds following the same procedure as described earlier. Two micrograms of total RNA from the sample was used for first-strand cDNA synthesis using an iScript cDNA synthesis kit (Bio-Rad, CA, USA). The reaction was performed in 20 μl reaction using 4 μl of 5X iScript reaction mix, 1 μl of reverse transcriptase, 7 μl of nuclease-free water, and 8 μl of RNA template. The complete reaction mix was incubated in a thermal cycler (BioRAD, CA, USA) under the following conditions: 25°C for 5 min, 42°C for 30 min, and 85°C for 5 min. RT-PCR primers were designed for 29 selected genes and maize *Actin 1* gene using Primer 3.0 software (Table 1). Maize *Actin1* (GRMZM2G126069) was used as an internal control for the normalization of gene expression. Each RT-PCR reaction was performed in a 25 μl reaction volume with following composition: 9.6–11.3 μl of nuclease free water; 5.0 μl of Promega Flexi green buffer; 2.5–4.0 μl of MgCl_2_(25 mM); 1.25 μl of each forward and reverse primer (50 ng/μl); 2.5 μl of dNTPs (2 mM); 0.2 μl of Promega Taq (5 U/μl); and 1.0–1.2 μl of template cDNA. PCR amplification was done using the following thermal cycling parameters: 95°C for 5 min; 95°C for 45 s; 55°C for 45 s; 72°C for 1 min, 35–40 cycles, and final extension at 72°C for 5 min. Equal amount of PCR reaction (20 μl) from each selected gene was electrophoresed in a 2.0% agarose gel in 1X TAE buffer. The ethidium bromide stained gels were visualized under a Kodak Gel Logic 200 Imaging system (Eastman Kodak Company, Rochester, NY, USA).

**Table 1:** **List of the genes and primers used for screening various maize germplasms using semi-quantitative RT-PCR**.

**S. No**	**Clone ID^a^**	**Locus name**	**Name of the gene**	**Sequence (5′–3′)**
1	P1A01	GRMZM2G154747	Plasma membrane associated protein [*Zea mays*]	F:TCTACGTGCTCATGCTCCAC
				R:TTGACACCACGACCTCATGT
2	P1A03	GRMZM2G116885	Cyclin-dependent protein kinase inhibitor 1	F:GGCATGGAATGGAACTCACT
				R:CAGGTGCAACATCACAGAGG
3	P1A12	GRMZM2G099860	Trehalose-6-phosphate synthase [*Zea mays*]	F:ACCTATGCAGCGGGTATGAC
				R:CTCCTTCATCGACACAAGCA
4	**P1B05**	GRMZM2G469523	Leucine rich repeat family protein	F:TGACGGACATATTGCTGCAT
				R:TTACCATTCGGCTTGAGGTC
5	P1B06	GRMZM2G090422	Xylose isomerase [*Zea mays*]	R:ATAGCCAAGGAGCTGCTCAA
				R:CCAGGCTGTAGCCTCAGAAC
6	**P1B07**	GRMZM2G117942	Pathogenesis-related protein 4 [*Setaria italica*]	F:TGACAGTCGGCAATAAGCTG
				R:CGCAGTTGACGAACTGGTAG
7	P1D06	GRMZM2G037683	WD40 repeat-containing protein SMU1-like	F:ACCTCCTTGTCCGCACATAC
				R:ATGCCAGCATTTCAGAATCC
8	P1E12	GRMZM2G161154	Metallothionein-like protein type 2 [*Zea mays*]	F:ACCACCACCCAGACTGTCAT
				F:AAGGCGATGGAGCAGATAGA
9	P1G07	GRMZM2G134797	Nucleoside diphosphate kinase 1 [*Oryza sativa*]	F:CATCATCAGTCGCTTCGAGA
				R:CATCCAAGCTCAACTGCTCA
10	P2B09	GRMZM2G091006	BETL-9 protein precursor [*Zea mays*]	F:TGCTGCTGAAACTGATTTGG
				R:CGAGTGGTGTTCTTGCTTGA
11	P2C09	GRMZM2G067919	Globulin-1 S allele precursor [*Zea mays*]	F:TCGGGTTCTTCTTCTTCCAA
				R:TTCACCTGCCTGTAGCTCCT
12	P2C12	GRMZM2G422651	Eukaryotic translation initiation factor 3 subunit 7	F:AAGTCGCGGAACTTCTTCAA
				R:GCAACCAAGCTTGTCTCCTC
13	P2D10	GRMZM2G169927	Activator of 90 kDa heat shock protein ATPase	F:GAACAGCTATCCTGCCCAAG
				R:CCAGGTGACTGAAGCTGTGA
14	P2E04	GRMZM2G125032	Putative 23S ribosomal RNA	F:CGCCAACGTGTACCCTTACT
				R:GCTTGTTGCCGTAGAAGAGG
15	P2E05	GRMZM2G173563	CP protein	F:AAGGTGGGGAGCTACTGGTT
				R:CCTGCAGTGAAATGGACCTT
16	**P3C05**	GRMZM2G362850	DEAD-box RNA helicase	F:CGCAAATGCAAAGTTCTCAA
				R:AACGATCACAGGGACTCCAC
17	P3F03	GRMZM2G061135	S-adenosylmethionine synthetase 1 [*Zea mays*]	F:CATGTTTGGGTATGCGACTG
				R:CACCGTAGGTGTCGATGATG
18	P4A02	GRMZM2G134806	Hsc70-interacting protein [*Zea mays*]	F:GTGATGCCAATGCTGCTCTA
				R:CTCTTGGCCTTGTCGTAAGC
19	P4E09	GRMZM2G399284	Chaperonin [*Zea mays*]	F:TCTTCGGCTTGGCTACAGTT
				R:AAACCCTGGACAAGTTCGTG
20	P4F10	AC233879.1_FG002	Embryonic abundant protein 1 [*Zea mays*]	F:CAGGTCAGGAAAGCAGGAAG
				R:ACTTGGACTCGTCGATGCTG
21	**P5A05**	GRMZM2G064136	Cation transport regulator-like protein 2 isoform	F:TTCGTTTCCACTCCAGATCC
				F:CAGACGATACAGCCTCACGA
22	P5B07	GRMZM5G870752	60S ribosomal protein L7a [*Zea mays*]	F:CGATCGAACTTGTTGTGTGG
				R:AGAAGCAAAGCTGCATGGAT
23	**P5C09**	GRMZM2G057642	RNA-binding protein 25 [*Zea mays*]	F:GACTATCCCATGGACCGAGA
				R:ACAGGTCTGTAACCGCCAAC
24	P5D12	GRMZM2G081464	Lipid transfer protein [*Oryza sativa*]	F:TTAGCACTCGCAGGTCACAG
				R:CGGATGTAGCTCCCGTAGTT
25	P5F05	GRMZM5G877316	Basic leucine zipper W2 domain-containing protein	F:TCTGGTTGCCAAAGGGATAG
				R:CTCGTGCAAAAAGCATTCAA
26	**P5G05**	GRMZM5G833124	Ubiquitin C-terminal hydrolase	F:GAGCAAGATTGTGTGGAGCA
				R:AAACAAATCAGCCGATGACC
27	−	GRMZM2G126069	Actin1	F:TGACCTCACCGACCACCTAA
				R:CCAGGGACGTGATCTCCTTG

a *Clones in bold font were used for qRT-PCR*.

### Validation of selected genes in germplasms by quantitative RT-PCR (qRT-PCR)

Total RNA isolated from all inbreds at two time points and control was used for qRT-PCR. Based on the metabolic functions and the results of RT-PCR, six genes were selected for further validation. cDNA was synthesized using 2 μg of total RNA using an iScript cDNA synthesis Kit (BioRAD, CA, USA) as described earlier. One microliter of cDNA was used for quantitative analysis of the gene expression. Expression of each gene was determined in three biological replications at each time point with three technical replicates. The reaction conditions consisted of the following steps: 95°C for 3 min; 40 cycles of 95°C for 30 s; 60°C for 30 s. Reactions were conducted in a MyiQ BioRad single color real-time PCR Detection system (BioRAD, CA, USA). Comparative CT method (2^−ΔΔCT^) was used to compute the fold changes in gene expression (Livak and Schmittgen, 2001). The list of primers used in qRT-PCR analysis and data generated from this experiment were given in Table S1 and Table S3, respectively.

### Co-localization of expressed genes on linkage map

DEGs from the SSH library were used for *in-silico* mapping and co-localization with the QTL for aflatoxin resistance on the linkage map. The sequences of these genes were submitted to Maize GDB Blast (http://maizegdb.org/popcorn/search/sequence_search/home.php?a=BLAST_UI) to search for genes with the highest similarity against the maize reference genome (B73 Ref Gen_v2MGSC). The bin position and physical location of these genes were obtained using the blast search in the maize genome database. The physical locations of linked markers with the QTL were obtained by using the locus lookup tool against the reference maize genome (Andorf et al., 2010). Using the physical location of the expressed genes and linked markers, the DEGs were placed on the linkage map constructed in the B73 × Mp715 F_2:3_ mapping population (Dhakal et al., 2016). In addition, two stable QTL identified in the Mp313E × B73 population (Brooks et al., 2005) on chromosome 2 (bin 2.05) and 4 (bin 4.08) were also included in this *in-silico* mapping. Co-localization was inferred using the information about the location of expressed genes and QTL location on the linkage map.

### Protein-protein interaction network analysis

Protein-protein interaction network of highly expressed important genes were analyzed using the publicly available program STRING (Search Tool for the Retrieval of Interacting Genes/Proteins) v 10 (Szklarczyk et al., 2015). It is a database of known and predicted protein-protein interactions that is widely used to analyze the network analysis and functional association between proteins on global scale. The amino acid sequences of the genes were obtained from phytozome using BLAST against maize proteome (https://phytozome.jgi.doe.gov) and queried against maize in STRING website (http://string-db.org) to detect all possible interactions.

## Results and discussion

### Aflatoxin accumulation in maize inbreds

Aflatoxin accumulation in maize inbreds was measured in 2013 and 2014. There was significant difference among the inbreds (Table 2). Va35 recorded the highest amount of aflatoxin in both years followed by B73, which indicated that both lines were highly susceptible to *A. flavus* infection as reported earlier (Brooks et al., 2005; Willcox et al., 2013). Mp715 and Mp313E, which showed the least amount of aflatoxin accumulation in both years, are well-known resistant inbred lines (Brooks et al., 2005; Warburton et al., 2011). Mp420 and Mp719 also showed lower aflatoxin accumulation (Williams et al., 2003). The resistant reaction of Mo18W was moderate as it showed relatively higher aflatoxin accumulation compared to the other resistant germplasm.

**Table 2:** **Mean aflatoxin (log transformed) and raw aflatoxin (ng g^**−1**^) accumulation in different maize germplasms during 2013, 2014, and across the years**.

**Inbred**	**Aflatoxin (log-transformed)^*^a^*^**			**Aflatoxin (ng g-1)**		
	**2013**	**2014**	**Across year**	**2014**	**2013**	**Across year**
B73	7.13^ab^	7.45^a^	7.29^a^	1347^b^	1780^a^	1563^b^
Mp715	4.15^c^	4.98^b^	4.57^c^	74^c^	162^c^	118^d^
Mp313E	4.18^c^	4.72^b^	4.45^c^	67^c^	118^c^	93^d^
Mo18W	6.35^b^	7.02^a^	6.69^b^	590^c^	1133^b^	862^c^
Va35	7.85^a^	7.62^a^	7.73^a^	2633^a^	2067^a^	2350^a^
Mp420	4.73^c^	4.82^b^	4.78^c^	135^c^	133^c^	134^d^
Mp719	4.56^c^	4.16^b^	4.36^c^	113^c^	73^c^	93^d^
Mean	5.56	5.82	5.69	709	781	745
SE	0.33	0.26	0.20	222	152	145
CV (%)	25.8	27.2	24.8	109.2	137.2	121.4

a *Values with same letter in the column did not differ significantly at P = 0.05*.

### SSH library, sequence assembly, and EST annotation

**Figure 1:**
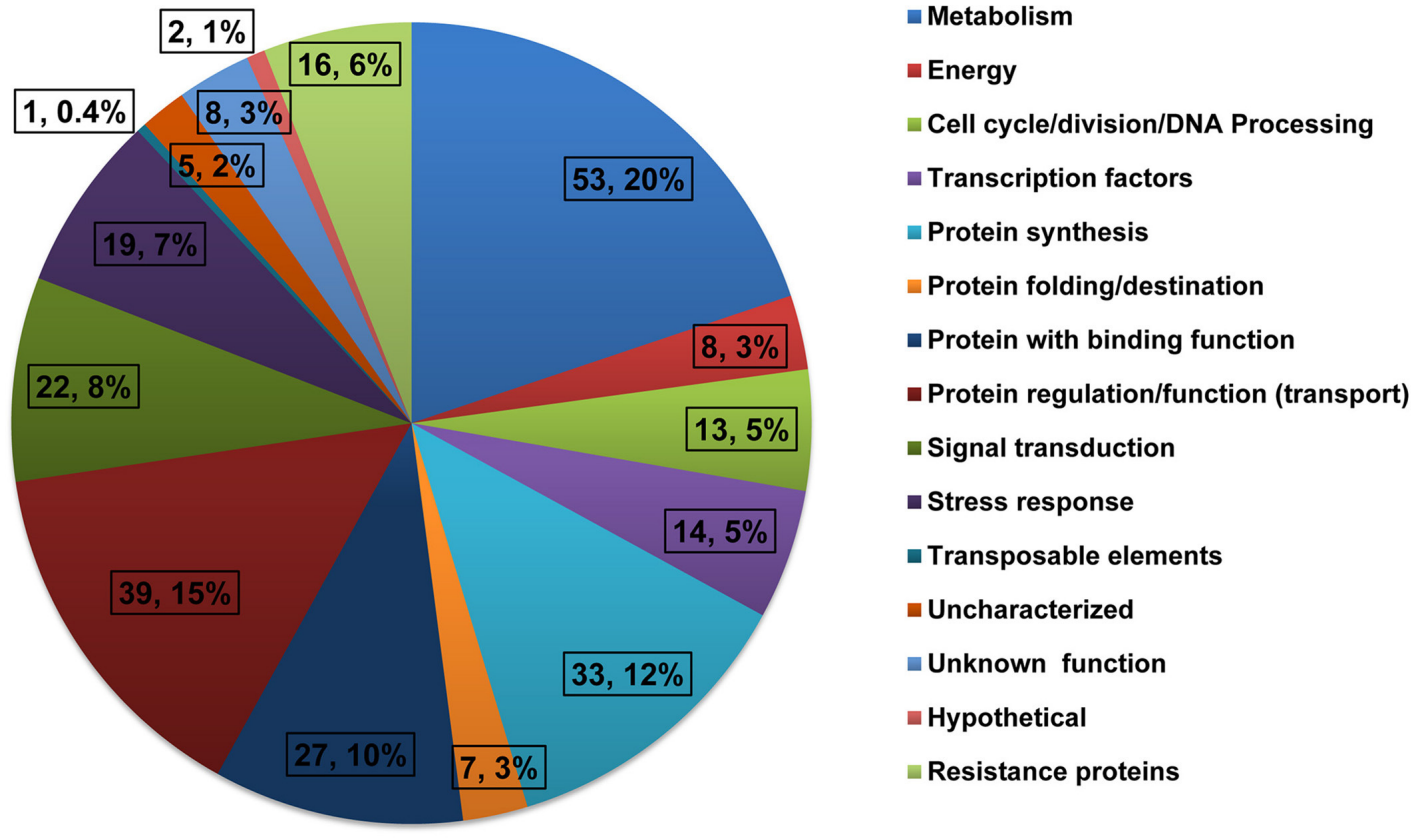
**Functional classification of differentially expressed genes from the SSH library constructed using developing ears of B73 (driver) and Mp715 (tester) after inoculation with ***A. flavus*****.

The quality of inserts was confirmed by PCR amplification and clones with single insert were used for sequencing. The clones with either repetitive or extremely short sequence (<80 bp) were excluded from further analysis. The remaining high quality sequences were used in homology search against the non-redundant protein database of the GenBank using BLASTX in NCBI (http://blast.ncbi.nlm.nih.gov/Blast.cgi). In total, 267 unique expressed sequence tags (ESTs) were identified and submitted to GenBank dbEST (www.ncbi.nlm.nih.gov/dbEST) with the accession numbers JZ969981-JZ970247 (Table S2). These sequences showed high similarities to nucleotide sequences from maize and represented genes of different biological functions. These ESTs were then classified into 15 different classes based on their biological functions (Figure 1). The groups with the highest number of ESTs were related to metabolism (20%), protein regulation/function (15%), and protein synthesis (12%). The other groups included in the library were ESTs related to signal transduction (8%), stress response (7%), and resistance proteins (6%).

### Identification of genes responding to *A. flavus* infection

**Figure 2:**
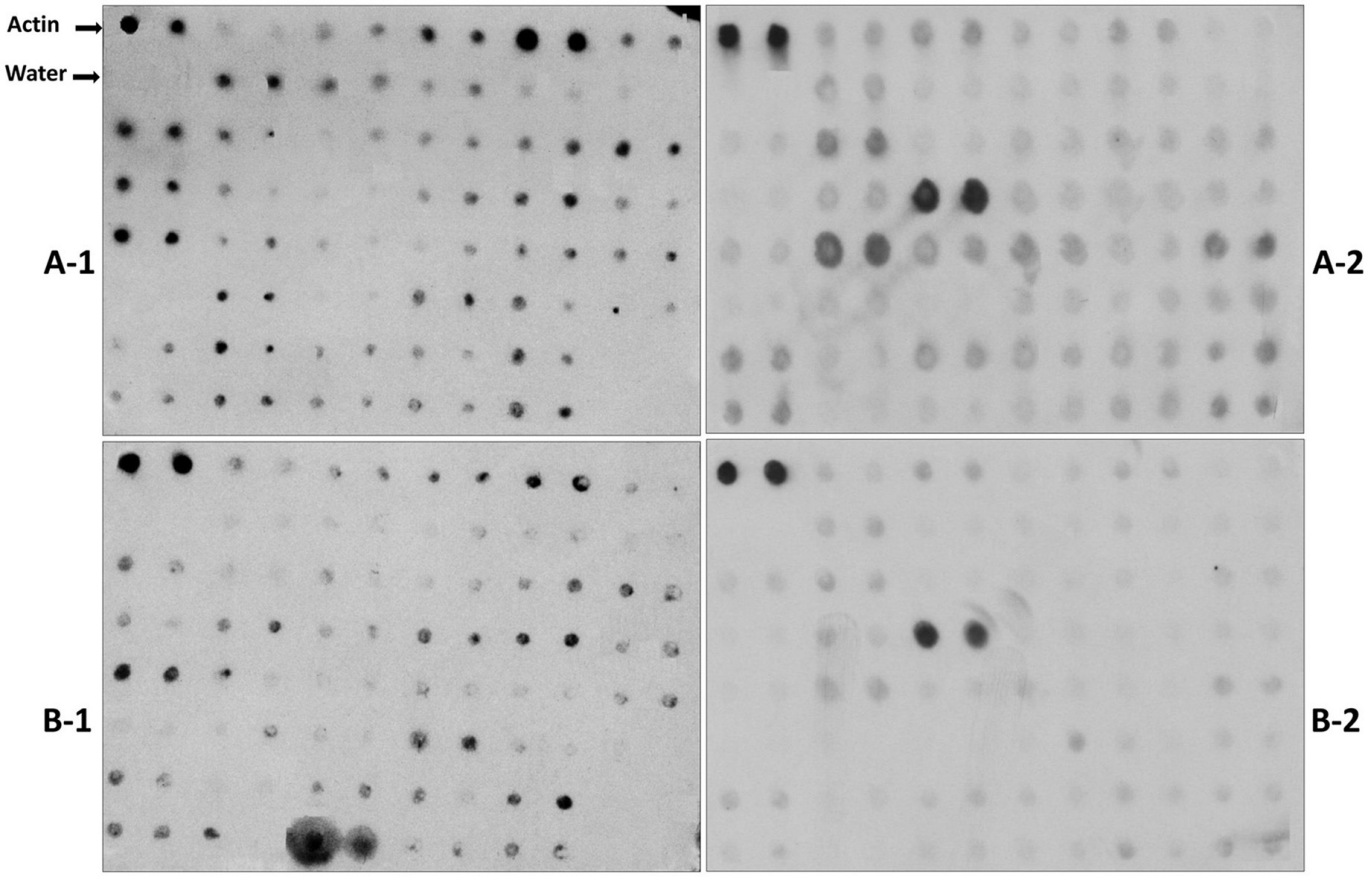
**Representative differential screening of the forward SSH library using reverse northern hybridization**. The PCR products of each cDNA clone were replicated twice on nylon membranes and the blots were hybridized with labeled probes. A-1 and A-2 are filters hybridized with the probe prepared from the inoculated Mp715 tissue. B-1 and B-2 are filters hybridized with B73 probe prepared from the inoculated tissue.

Reverse northern hybridization was performed to further confirm the expression of the differentially expressed clones from the unique cDNA clones (Table S2) using labeled cDNA probes of the resistant inbred Mp715 and the susceptible inbred B73. Hybridization blots with different probes (Figure 2) showed strong hybridization signals for most of the clones when probed from inoculated Mp715 cDNA whereas hybridization signal was weak or absent when probed with inoculated B73 cDNA. Since Mp715 is a resistant line, clones with strong hybridization signals with Mp715 were considered as good candidates for aflatoxin resistance. Characterization of the up-regulated genes would be helpful for exploring the molecular mechanisms of aflatoxin resistance in maize. Twenty-six DEGs were selected for expression analysis using semi-quantitative RT-PCR.

### Genes involved in carbohydrate metabolism

Transcriptional changes in various genes involved in carbohydrate metabolism occur in developing maize kernels after *A. flavus* infection. A large number of genes involved in the synthesis and hydrolysis of starch and the mobilization of sugars were identified (Table S2). Genes with high similarity to UDP-sugar pyrophospharylase, asparagine synthetase, transaldolase, trehalsoe-6-phosphate synthase, fructose-6-phosphate, phosphoglyceromutase, xylose isomerase, fructose-1,6-bisphosphatase, putative oxidoreductase, phosphofructokinase, pyrophosphate-fructose -6-phosphate 1-phosphotransferase, glutamine-dependent NAD (+) synthetase, glyceraldehyde-3-phosphate dehydrogenase, acetaldehyde dehydrogenase, acetyl-CoA dehydrogenase, ADP-glucose pyrophosphorylase embryo small subunit, shikimate kinase, and acyl-thioesterase were highly expressed in reverse northern hybridization with inoculated Mp715 probe. In plants, although carbohydrates mainly act as a substrate for growth and development, it is not directly associated with resistance. However, changes in carbohydrate metabolism affect the sugar sensing system and initiate the transcription of defense related genes (Koch et al., 1995; Bolton et al., 2008). Changes in the expression of genes involved in the glycolytic pathway occur in response to infection. The hexoses are transported to the cell to provide energy and carbon for initiating resistance response (Bolton et al., 2008). Dolezal et al. (2014) reported upregulation of phosphofructokinase (PFK) in maize kernels in response to *A. flavus* infection. These hexose kinases are associated with sugar sensing and regulate programmed cell death in plants when pathogen attacks occur (Kim et al., 2006; Granot et al., 2013). Further proof of the role of carbohydrate metabolism in eliciting defense response came from a study in wheat in which upregulation of PFK and PPi-PFK in response to *Puccinia triticina* was reported (Bolton et al., 2008). Likewise, fructose-6-phosphatase, which was highly upregulated in our study, is involved in starch synthesis for the generation of defense-related compounds (Dolezal et al., 2014). Expression of shikimate kinase and chorismate mutase in our study suggests that these may be involved in host plant defense (Daayf and Lattanzio, 2009). Chorismate is utilized to synthesize aromatic amino acids, which are precursors to several flavonoids and phytoalexins. It also provides the precursors for lignin biosynthesis, a major cell wall component involved in the basal resistance to pathogen (Herrmann, 1995). Higher lignin content was observed in resistant varieties of maize (Luo et al., 2011; Kelly et al., 2012) and peanut (Liang et al., 2006) after *A. flavus* infection. Zein-alpha 19C2 precursor (P1B10) and various acetyl CoA derivatives, which were highly expressed in our experiment, are involved in biosynthetic and metabolic pathways (Kelly et al., 2012; Wang et al., 2013). All these studies along with our findings suggest that carbohydrate metabolism related genes play important role in host plant resistance against *A. flavus* in maize.

### Genes associated with signal transduction pathway

Genes related to signal transduction were highly expressed in response to *A. flavus* infection. These are mainly plant receptor protein kinases (RPK), which are involved in sensing the pathogen signals and the acceleration of inducible defense (Garcia-Brugger et al., 2006). Mitogen activated protein kinase (MAPK) cascades are extremely important for the regulation of signaling pathways against abiotic and biotic stresses (Rodriguez et al., 2010). These signaling cascades play important role in regulating cross-talk between stress responses (Andreasson and Ellis, 2010). ESTs highly similar to protein kinases, mitogen-activated protein kinases, and CBL interacting proteins were highly expressed when probed with cDNA from inoculated resistant inbred MP715. Genes highly similar to leukocyte receptor cluster membrane proteins, CBL-interacting protein kinases, serine/threonine protein kinases, flower specific gamma-thionin precursors, mitogen-activated protein kinase 7, plasma membrane associated proteins, and protein tyrosine phosphatases were differentially expressed in the library. Further confirmation was done by reverse northern analysis. The increased levels of Ca^2+^ in the host plant in response to pathogen elicitors are responsible for increased resistance in plants through the activation of calcium-dependent kinases, which include calcineurin B-like proteins (CBL) that induce downstream genes (Tena et al., 2011; Asano et al., 2012). Differential expression of kinases and CBL-interacting protein in our SSH library indicated increased level of Ca^2+^ in the kernel after infection. Calcium-mediated immunity has been reported on host-pathogen interactions through protein kinases and CBL-proteins (Tena et al., 2011). Bedre et al. (2015) reported differential expression of these transcripts in cotton. Many signal transduction pathway genes were previously identified in response to *A. flavus* inoculation in maize (Jiang et al., 2011; Zhang et al., 2012). Identification of these genes in our experiment suggests their potential role in resistance reaction in maize plant.

### Transcription factors

Transcription factors control suppression or activation of downstream genes in response to pathogen invasion (Guo et al., 2011). Few transcription factor genes that were highly expressed in our library include elongation factor 1-delta, eukaryotic translation initiation factor 3, bZIP transcription factors, zinc finger protein binding protein, and zinc finger proteins (Table S2; Figure 2). Reverse northern experiment indicated upregulation of these genes in resistant inbred Mp715. Zinc finger binding proteins with a bZIP domain(s) regulate many genes involved in response to abiotic stress and pathogen infection (Jakoby et al., 2002). These genes were expressed at a higher level in maize leaf sheath (Gao et al., 2014) and kernels (Zhang et al., 2012) inoculated with *Rhizoctonia solani*. The zinc finger proteins were induced after fungus inoculation in cotton (Bedre et al., 2015).

### Genes involved in stress response

Several stress response genes were known to be expressed in the host-plant after pathogen attack (Cleveland et al., 2003). The genes showing similarity to late embryogenesis abundant protein, Win1 precursor, protein phosphatase 2C, wound-induced protein WIN2 precursor, cysteine proteinase inhibitor, Dnaj heat shock protein, chaperone protein, Bowman-Birk type wound induced proteinase inhibitor, globulin-1 S allele precursor, metallothione-like protein, cystatin, and stromal ascorbate peroxidase were represented in our SSH library (Table S2). Expression of these genes was higher in Mp715 compared to B73 after the inoculation of *A. flavus* (Figure 2). Heat shock proteins (HSPs) are often expressed in response to drought, pathogen infection, and wounding. HSPs serve as molecular chaperones due to their role in the stabilization of the denatured proteins and proper protein folding (Bartels and Sunkar, 2005). Three HSPs highly expressed in resistant inbreds were known to be involved in heat and drought stress response in maize and wheat (Hu et al., 2010) and were highly expressed in the seed of resistant maize inbreds (Luo et al., 2011; Kelly et al., 2012; Asters et al., 2014), and cotton (Bedre et al., 2015).

Oxidative burst is the earliest response against pathogen infection and is associated with reactive oxygen species (ROS), hypersensitive reaction, and programmed cell death (Wang et al., 2010). ROS are involved in killing pathogens and in activating defense cascades (Dolezal et al., 2014). Peroxidases are associated with this defense response, and two peroxidase-annotated transcripts were highly expressed in resistant inbreds in our study which concur with the observation of Dolezal et al. (2014). These were downregulated under stress, leading to higher aflatoxin content in maize (Luo et al., 2010). Downregulation of peroxidase may create conducive environment for fungus infection on host plant and subsequent production of aflatoxin in seed by delaying the pathogen recognition, signaling cascade initiation, and killing of pathogen. Two wound inducible genes, such as Bowman-Birk like proteinase and wound induced protein WIN2 precursor, reported by Rohrmeier and Lehle (1993), were highly expressed in response to fungal inoculation in our experiment. Higher expression of stress responsive genes in our study further support their roles in disease resistance and these could be useful for improving host plant resistance to reduce aflatoxin accumulation in maize.

### Genes associated with disease resistance

Plants depend on the innate immune system for recognition and response to potential pathogens. Plants synthesize low molecular proteins and peptides with antifungal activities (Selitrennikoff, 2001). Pathogenesis-related (PR) proteins increase host plant resistance against pathogens (Wang et al., 2014). PR proteins are disease resistance genes induced in the host plant in response to pathogen infection (Muthukrishnan et al., 2001; Bravo et al., 2003; Luo et al., 2011). Sixteen disease resistance genes were differentially expressed (Table S2). The PR-protein genes include PR-1, PR-4, PR-5, PR-10, and chitinase. Reverse northern experiment with inoculated probe of Mp715 showed higher expression of PR-4 (Figure 2). In addition to these PR proteins, other differentially expressed disease resistance genes include multidrug resistance protein, vacuolar defense protein, leucine rich repeat (LRR) family protein, and Barperm1. PR-proteins from plants were well studied and characterized and transgenic expression of these genes exhibited increased resistance against various fungi (Muthukrishnan et al., 2001). PR-1 was upregulated transgenic maize plants after the inoculation of *Exserohilum turcicum* (Wang et al., 2014). The PR-5 protein causes cytoplasmic leakage and hyphal rupture of fungus and reduces the fungal infection. Higher expression of PR-5 was also observed in maize silk (Sekhon et al., 2006) and maize kernels (Jiang et al., 2011; Wang et al., 2014; Shu et al., 2015) after the inoculation of fungus. PR-1 and PR-5 were highly induced in response to pathogen attack in maize (Morris et al., 1998). Chitinase belongs to the PR-3 family, and its induction in response to fungus inoculation has an important role in host resistance. Most fungi contain chitin in their cell wall and the hydrolytic activity of chitinase weakens the cell wall (Selitrennikoff, 2001). The increased protection by chitinase is due to direct inhibition of fungal growth and induced defense response of Glc-NAc oligomers produced by their activity (Ham et al., 1991). Several studies showed higher expression of PR proteins in *A. flavus* and *F. verticilloides* infected maize kernels (Dolezal et al., 2014; Shu et al., 2015). Therefore, manipulation of PR- proteins in maize kernels may result in enhanced resistance against *A. flavus* in maize.

### Gene expression analysis in maize germplasms by semi-quantitative RT-PCR

**Figure 3:**
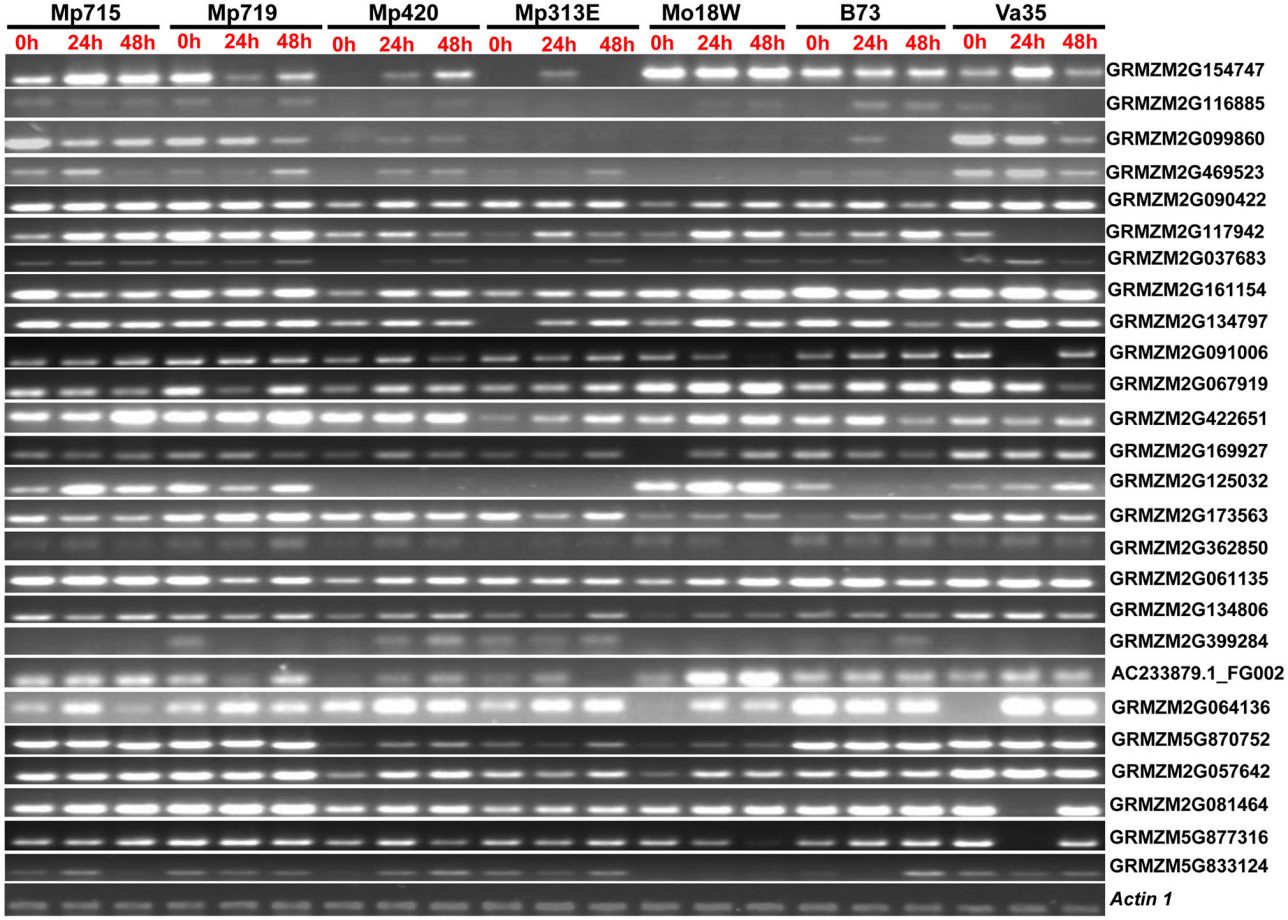
**Expression pattern of the 26 selected genes from the SSH library of ***Zea mays*** in different inbred lines with variable resistance to aflatoxin accumulation at different time points after ***A. flavus*** inoculation in semi-quantitative RT-PCR analysis**. Locus names on the right side of the figure correspond to the genes listed in the Table 1. Detail information about these genes is provided in Table S2. *Actin 1* is used as internal control.

To validate the expression patterns of the genes selected from reverse northern experiment, semi-quantitative RT-PCR was used for selected genes potentially involved in important physiological pathways leading to disease resistance. Altogether, 26 highly expressed genes were analyzed at three different time points (0, 24, and 48 h) after inoculation of *A. flavus* (Figure 3; Table 1). These genes showed differential expression patterns at different time points in resistant and susceptible germplasm. Genes associated with disease resistance, such as PR-4 (GRMZM2G117942), leucine rich repeat family protein (GRMZM2G469523), and DEAD-box RNA helicase (GRMZM2G362850), were highly expressed in resistant inbreds compared to susceptible inbreds. The expression of PR-4 increased with time in resistant inbreds but decreased in the susceptible inbred Va35. Expression of LRR family proteins increased with time after inoculation but was extremely low in Mo18W and B73. Its expression decreased along with time in Va35. Genes involved in carbohydrate metabolism [trehalose-6-phosphate (GRMZM2G099860)] was highly induced in resistant germplasms. Embryonic abundant protein (AC233879.1_FG002), which is related to stress, was highly expressed in resistant inbreds compared to susceptible inbreds (B73 and Va35). Some transcription factors also exhibited higher expression in resistant germplasm. Overall, RT-PCR analysis confirmed the expression patterns observed in reverse northern experiment. Since the genes involved in disease resistance, stress response, and signal transduction were highly expressed in all resistant inbreds compared to susceptible inbreds, these genes could be involved in protecting maize against *A. flavus* infection.

### Quantitative real-time RT-PCR (qRT-PCR) for gene expression in germplasms

**Figure 4:**
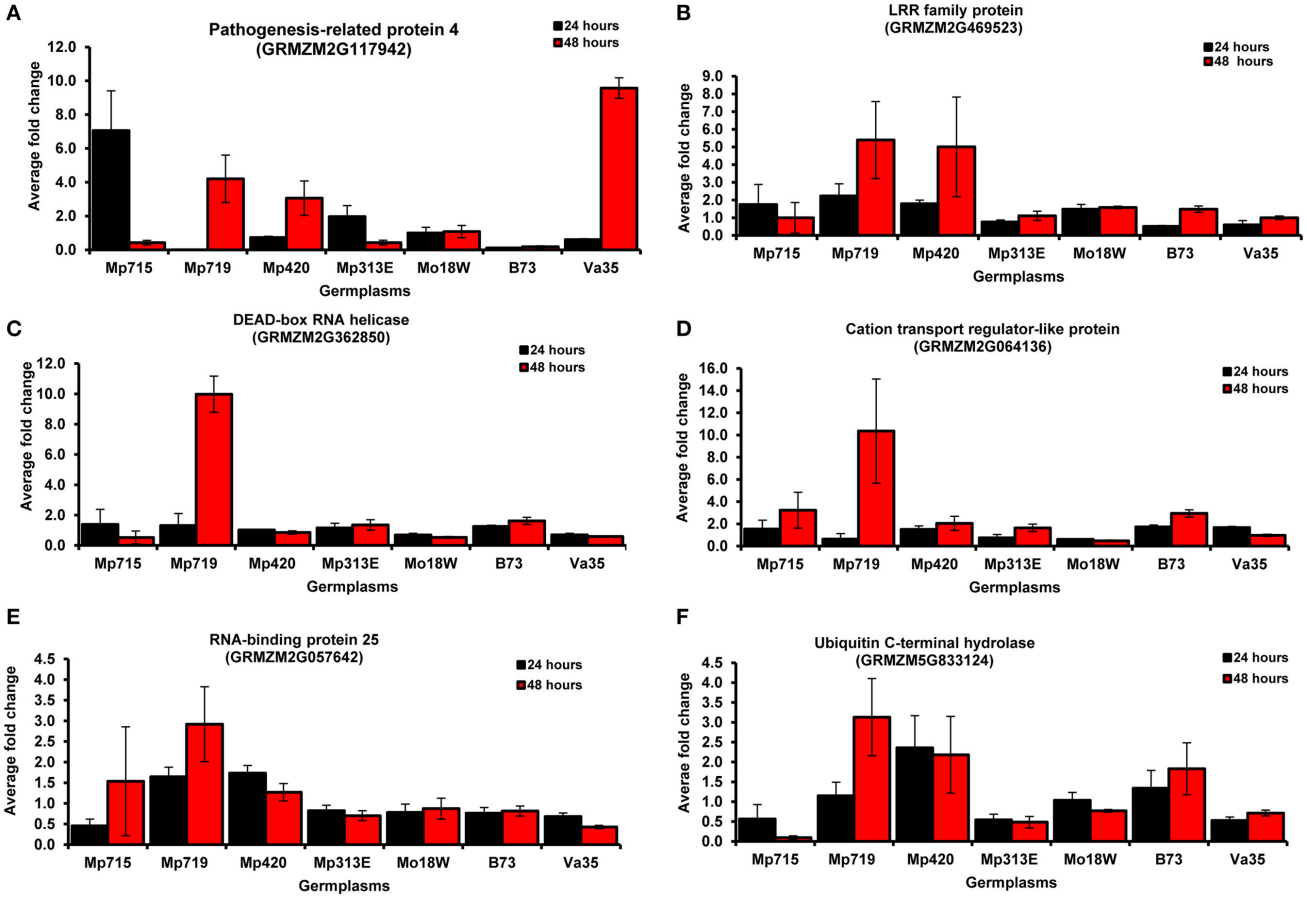
**Real-time RT-PCR (qRT-PCR) analysis of six genes selected from the SSH cDNA library**. Expression of these genes was evaluated at two time points (24 and 48 h after inoculation) in different maize germplasms using *Actin 1* as an internal standard. The average fold change in expression was compared with control in each inbred line. **(A)** pathogenesis-related protein 4 (PR 4); **(B)** Leucine rich repeat family protein; **(C)** DEAD-box RNA helicase; **(D)** Cation transport regulator-like protein 2 isoform; **(E)** RNA binding protein 25; and **(F)** Ubiquitin C-terminal hydrolase.

After the evaluation of expression patterns of selected genes through semi-quantitative RT-PCR, six important genes were selected for qRT-PCR to further validate their expression patterns (Figure 4, Table S3). All selected genes were highly expressed at 48 h after infection (HAI) in Mp719 than in other inbreds. However, the genes, PR-4, LRR family protein, RNA binding protein, and ubiquitin C-terminal hydrolase were highly expressed in resistant inbreds. One exception is that PR-4 was highly expressed (10-fold) in susceptible inbred Va35 at 48 h after inoculation compared to the control, but extremely low in B73 (0.2-fold). In other resistant inbreds such as Mp719 and Mp420, its expression increased along with time, and was 4 and 3 times higher at 48 HAI compared to their respective control. It exhibited highest expression in Mp715 and Mp313E at 24 HAI and decreased afterwards. Likewise, LRR family protein was highly expressed in B73 at 24 HAI compared to Va35, but their expression was low compared to other resistant inbreds. Expression patterns of the LRR family proteins showed similar patterns as PR-4 in Mp715, Mp719, and Mp420. PR-4, DEAD-box RNA helicase, and LRR repeat family protein responded quickly to fungus inoculation, and their expression reached to maximum at 24 HAI and decreased afterwards. Cation transport regulator-like protein expression increased along with time after fungal inoculation in resistant inbreds. Highest expression (10-fold) was observed in Mp719 at 48 HAI compared to control. In contrast, its expression reduced in Va35 as time advanced after inoculation. Similarly, RNA binding protein 25 (GRMZM2G057642) and ubiquitin C-terminal hydrolase (GRMZM2G833124) were highly expressed at 24 and 48 HAI in Mp719 and Mp420 compared to other inbreds. Their expression in Mp719 increased along with time and was 3 times higher at 48 HAI compared to their respective control. However, the expression of these two genes reduced slightly at 48 HAI in Mp420 compared to 24 HAI. The expression pattern of the selected genes in qRT-PCR was in agreement with the results of RT-PCR.

The pathogenesis related proteins (PR-1, PR-5, PRm3, and PRm6) exhibited high level of expression in resistant inbreds after fungal inoculation of maize kernels (Jiang et al., 2011; Luo et al., 2011; Kelly et al., 2012; Asters et al., 2014; Shan and Williams, 2014; Alessandra et al., 2015), and silks (Sekhon et al., 2006). Similarly, Chen et al. (2006) reported increased expression of PR-10 in resistant germplasm GT-MAS:gk, and susceptibility to *A. flavus* was increased by repression of maize PR-10 gene by RNAi (Chen et al., 2010). The defense response and signal transduction pathways play important role in host-pathogen interactions (Tarchevsky, 2001). They are responsible for basal and broad-spectrum pathways related to defense against environment and pathogen (Durrant and Dong, 2004). Proteomic studies in maize rachis also revealed similar gene expression pattern in response to fungal inoculation (Pechanova et al., 2011). Similarly, the role of DEAD-box RNA helicase is well-established in RNA transport, translation initiation, RNA degradation, and gene expression in various organelle (Rocak and Linder, 2004). They are also involved in cellular differentiation, cell cycle, and responses to abiotic stress. Most of them act as a regulator of development processes and tolerance to abiotic stress like salt stress, and temperature (Owttrim, 2006). DEAD-box RNA helicase genes (*STRS1* and *STRS2* in *A. thaliana* and *OsABP* in rice) were highly responsive to abiotic stresses (Kant et al., 2007; Macovei et al., 2012). Recently, the involvement DEAD-box RNA helicase gene (*SIDEAD31*) in the regulation of drought tolerance and stress-related genes in tomato was reported (Zhu et al., 2015). These results demonstrate the importance and involvement of DEAD-box RNA helicase genes stress responses. Since aflatoxin accumulation in corn is exacerbated during grain filling under drought stress (Payne, 1998), this gene has potential to reduce pre-harvest aflatoxin accumulation by improving drought tolerance. Aflatoxin accumulation was low in these resistant inbreds during field evaluation (Table 2). Upregulation of these genes in resistant germplasm in our study suggests that these genes can be targeted for manipulation to reduce aflatoxin accumulation in maize.

### Co-localization of differentially expressed genes with QTL for resistance to aflatoxin accumulation

**Figure 5:**
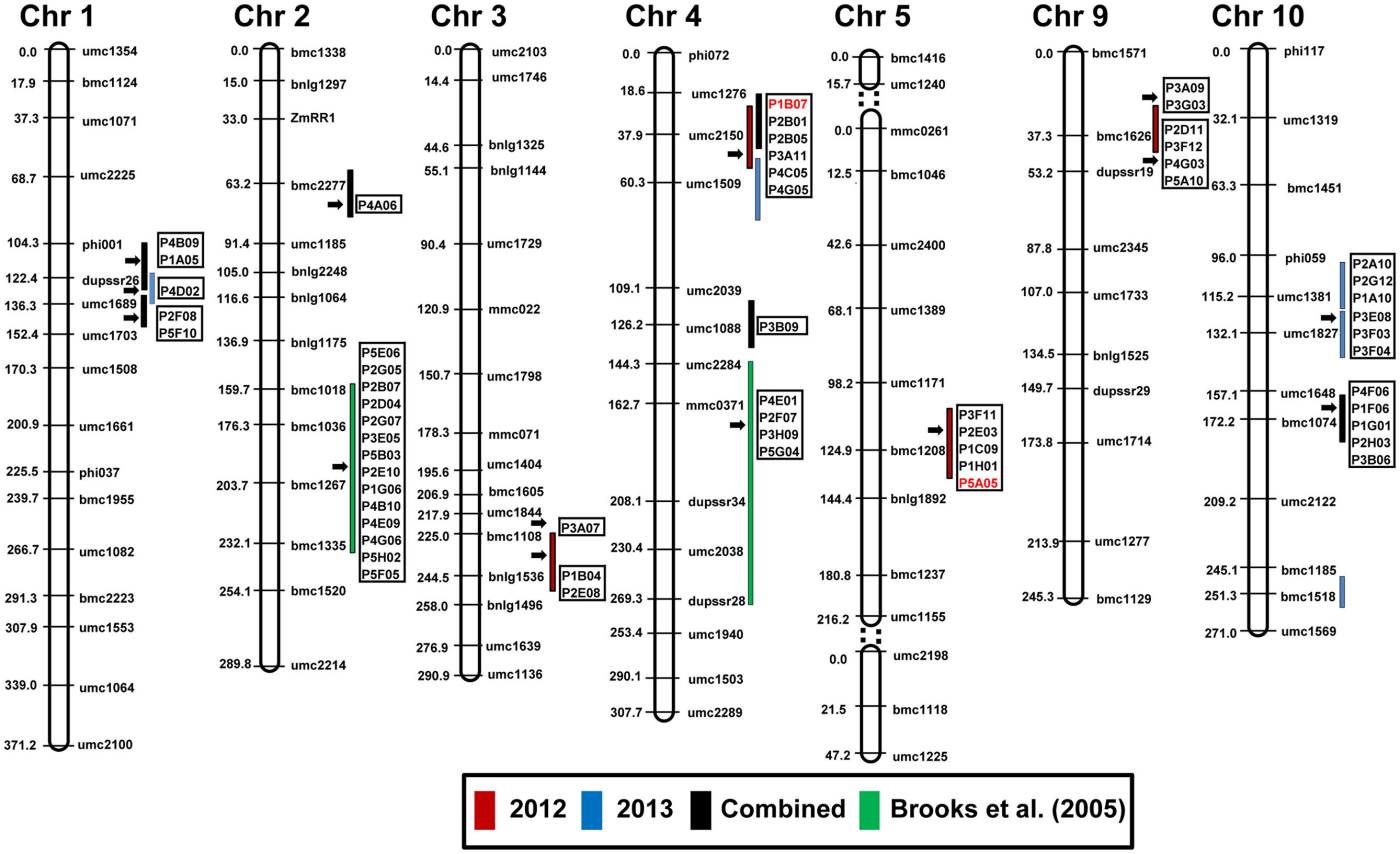
**Co-localization of differentially expressed genes from the SSH library with the QTL for resistance to aflatoxin accumulation in an ***F***_**2:3**_ population derived from B73 × Mp715 (Dhakal et al., **2016**)**. Red, blue, and black bars represent QTL for aflatoxin resistance during 2012, 2013, and combined analysis, respectively. Clone ID in red font were used in RT-PCR for expression studies on various inbred lines. Green bars represent the location of QTL identified by Brooks et al. (2005). Detail information about the genes is provided in Table S2.

The ESTs from the SSH library were mapped on to the maize linkage map constructed from the cross B73 × Mp715 (Dhakal et al., 2016; Figure 5; Table S2). Altogether 56 genes with various physiological functions co-localized with the QTL regions. These genes showed high similarity (>95%) to the annotated genes on the reference maize genome and were distributed on different chromosomes, except chromosomes 6, 7, and 8. Majority of these genes were mapped on/near QTL for resistance to aflatoxin accumulation on chromosomes 2, 4, 5, and 10. These genes were largely related to metabolism, stress response, and disease resistance suggesting the usefulness of this integrated approach to identify candidate genes to prevent *A. flavus* infection.

A close examination of the functionality of the ESTs mapped in the QTL regions revealed an interesting trend. Six ESTs belonging to resistance proteins category including PR-4 and win2 precursor, which were disease resistance and stress response genes, respectively, co-localized with the QTL for resistance to aflatoxin on chromosome 4 near umc2150 and umc1509. Higher expression of PR-4 was observed in resistant inbreds compared to B73 in response to *A. flavus* inoculation (Figures 3, 4). Similarly, five ESTs including the cation transport regulator -like protein overlapped with the QTL on chromosome 5, were related to metabolism. Cation transport regulator like-protein was highly expressed in resistant germplasms and confirmed by RT-PCR and qPCR.

Four ESTs with high similarity to lamar-type protein kinase, 60S ribosomal protein, phosphotransferase system, and shikimate kinase, respectively, were mapped near the previously identified QTL on chromosome 4 (Brooks et al., 2005). However, these belonged to different functional category. In case of the QTL on chromosome 2 identified by Brooks et al. (2005), several stress responsive and metabolism related genes were mapped.

The ESTs mapped near QTL on chromosome 9 included genes homologous to hemolysin family calcium-binding protein, ATP-dependent Clp protease proteolytic subunit 2, Cytochrome P450 family protein, 60S ribosomal protein L11, and elongation factor 1-alpha. Similarly, ESTs homologous to diphosphonucleotide phosphatase, aspartic proteinase, S-adenosyl methionine synthetase1, chitinase, and putative mitochondrial Rieske protein genes coincided with QTL for resistance to aflatoxin accumulation on chromosome 10.

### Protein-protein interaction networks

**Figure 6:**
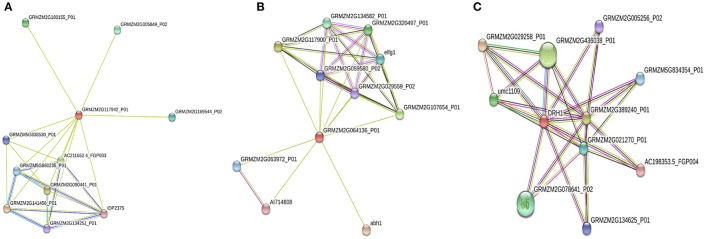
**Protein-Protein interaction analysis for the pathogenesis related protein-4 (GRMZM2g117942) (A)**, Cation transport regulator-like protein (GRMZM2G064136) **(B)**, and DEAD-box RNA helicase (DRH1) **(C)** in maize. Each node in the network represents protein produced by single protein coding gene. Small nodes: Protein of unknown 3D structure (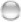); Large nodes: some 3D structure is known or predicted (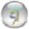); Colored nodes: query proteins and first shell of interactors (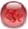). Each edge represents P-P association.

Protein-protein interaction is extremely important to identify and analyze the functional association in biological processes (Szklarczyk et al., 2015). Some genes involved in disease resistance were highly expressed and located on the QTL region identified in our mapping experiment. Protein-protein interaction analysis was performed using STRING software for PR-4 (GRMZM2G117942), cation transport regulator-like protein (GRMZM2G064136), and DEAD-box helicase (GRMZM2G362850), which were highly expressed in response of *A. flavus* inoculation. The interaction network analysis of PR-4 revealed its association with both known and uncharacterized proteins (Figure 6A; Table S4). Glycoside hydrolase and chitinase families were identified in both INTERPRO and PFAM pathway analysis. Glycoside hydrolases are known to be involved in degradation of complex sugars whereas the role of chitinase is well-established in fungal cell wall degradation and disease resistance (Selitrennikoff, 2001).

The interaction analysis of cation transport regulator-like protein, and DEAD-box RNA helicase (Figures 6B,C) revealed their involvement in translation, Glutathione S-transferase (GST), and *S-Adenosyl Methionine* pathways, respectively (Table S4). GSTs are involved in reduction of ROS during oxidative stress (Dixon et al., 2002). These genes interact with numerous hypothetical and uncharacterized maize genes, elongation factor gene, and heat shock protein binding protein (Table S5), which may help reduce aflatoxin accumulation through enhancement of abiotic stress tolerance. The upregulation of these genes in response to *A. flavus* infection was validated in resistant germplasm through RT-PCR/qPCR. The protein-protein interaction analysis further supported the role of these genes in improving disease resistance either directly inhibiting infection or indirectly improving abiotic stress tolerance. Therefore, further characterization of these genes involved in these processes will be helpful to understand their function and mechanism in host-plant resistance.

## Conclusion

Due to the genetic complexity and involvement of multiple environmental factors, a multipronged approach is necessary for sustainable management of aflatoxin accumulation in corn. Our earlier mapping study (Dhakal et al., 2016) identified QTL regions for resistance to aflatoxin on chromosome 4 (bin 4.01–4.02) and 10 (bin 10.03–10.05). *In-silico* mapping revealed overlapping of a number of genes involved in disease resistance, stress resistance, and metabolism with the QTL regions reported in our study as well as of others. These genes are potential candidates for future studies to understand host-plant resistance mechanisms and develop molecular markers for utilization in breeding program to develop aflatoxin resistant maize inbreds, and hybrids. Results from this study and other gene expression studies (Luo et al., 2010; Jiang et al., 2011; Kelly et al., 2012; Zhang et al., 2012; Dolezal et al., 2014) provide a solid foundation for investigation into host-pathogen interaction.

## Author contributions

RD and PS conceived and designed the experiment. RD, RK, and CC conducted experiments. RD analyzed and wrote the manuscript. WW, GW, PS, RK, and CC helped in editing the manuscript. All authors read and approved the manuscripts for submission.

### Conflict of interest statement

The authors declare that the research was conducted in the absence of any commercial or financial relationships that could be construed as a potential conflict of interest.

## Abbreviations

QTL: quantitative trait loci
SSH: suppression subtraction hybridization
EST: expressed sequence tags
DEG: differentially expressed genes
qPCR: Quantitative RT- PCR
MAB: marker assisted breeding
NCBI: National Center for Biotechnology Information
PR: Pathogenesis-related.
