# Supplementary material for: Expression Profiling Coupled with In-silico Mapping Identifies Candidate Genes for Reducing Aflatoxin Accumulation in Maize
Source: Front Plant Sci. 2017 Apr 6;8:503. doi: 10.3389/fpls.2017.00503 (PMC5382453; doi:10.3389/fpls.2017.00503)
Supplement: Supplementary file 1 [file Table1.DOCX]

**Table S1** List of the primers used for real-time RT-PCR (qRT-PCR)

| **Locus name** | **Name of the gene** | **Sequence (5'-3')** | **Product size (bp)** |
| --- | --- | --- | --- |
| GRMZM2G117942 | Pathogenesis-related protein 4 | F:TGACAGTCGGCAATAAGCTG | 118 |
|  |  | R:CTGCTGCGGGTTGTAGAAGT |  |
| GRMZM2G469523 | Leucine rich repeat family protein | F:GTACATTCGGTTGCGATGTG | 121 |
|  |  | R:TCTGCCACCGGCTCTATACT |  |
| GRMZM2G362850 | DEAD-box RNA helicase | F:TTTGGAAGGACCCTGTCTTG | 123 |
|  |  | R:CGCACACCAATAGAGAGCAA |  |
| GRMZM2G064136 | Cation transport regulator-like protein | F:TCGTGAGGCTGTATCGTCTG | 104 |
|  |  | R:TCCTCCAAGTTCAAGGATCG |  |
| GRMZM2G057642 | RNA-binding protein 25 [*Zea mays*] | F:GGCAACCGTATTGTTCGAGT | 137 |
|  |  | R:TCATAGCTCCGTTCCCTGTC |  |
| GRMZM5G833124 | Ubiquitin C-terminal hydrolase | F:TCATTTGCCTTCTTGTCGTG | 91 |
|  |  | R:TGAACCTTGCCCTATTCAGG |  |
| GRMZM2G126069 | Actin1 | F:GAAACCTTCGAATGCCCAGC | 105 |
|  |  | R:CACACCATCACCGGAATCCA |  |
